# Dynamics of cocoa fermentation and its effect on quality

**DOI:** 10.1038/s41598-021-95703-2

**Published:** 2021-08-18

**Authors:** Ana M. Calvo, Blanca L. Botina, Maria C. García, William A. Cardona, Andrea C. Montenegro, Jenifer Criollo

**Affiliations:** 1 Corporación Colombiana de Investigación Agropecuaria – AGROSAVIA, Tibaitatá Research Center – Km 14 Route Mosquera-Bogotá, Cundinamarca, Colombia; 2 Corporación Colombiana de Investigación Agropecuaria – AGROSAVIA, Nataima Research Center – Km 9 Route – Espinal-Chicoral, Tolima, Colombia

**Keywords:** Biochemistry, Plant sciences, Chemistry, Engineering

## Abstract

Several research efforts on cocoa have been focused on parameters for controlling the transformation process to guarantee homogeneity and quality of cocoa beans, the main raw material in the chocolate industry. The main changes that determine the final quality of cocoa—and also the product’s homogeneity—occur during fermentation, given the great number of factors that affect the process. This research seeks to identify the most relevant factors affecting quality in order to offer higher-quality and more homogeneous cocoa for the chocolate industry. The dynamics of the fermentation process were observed in three contrasting locations, monitoring different variables and evaluating the final quality of the cocoa. Results show that temperature and pH profile are the key factors to be monitored and controlled in order to achieve high-quality cocoa beans.

## Introduction

Cocoa is the most important and valuable input for the chocolate industry^1,2^, which faces a new challenge every day, seeking to maintain the supply of standard products with non-standardized raw materials^3^. Cocoa composition and quality are affected by different factors, such as the genetic profile of the material^4^, environmental conditions of the production site, state of maturity of the beans, and processing conditions^2,5–10^. Therefore, cocoa producers and processors must also face this challenge since there are no guiding parameters for either the raw material or the transformation processes to facilitate obtaining a homogeneous product of high organoleptic attributes. The sensory analysis, largely affected by the profile of volatile aromas, determines rejection or acceptability, and the lower or higher value of cocoa in the market. Developing this aromatic profile throughout the production and processing of cocoa is highly complex and depends, in addition to the factors already listed, on a series of biochemical reactions that take place inside the bean once the embryo dies due to the action of temperature and the entry of organic acids. These acids also break the cell’s membranes, allowing substrates and enzymes to mix, giving way to the oxidation of polyphenols, formation of tannins, catabolism of proteins, degradation of sugars, among many other changes. These acids and the increase in temperature that promote these changes inside the bean come from the fermentation of sugars and organic acids present in the pulp surrounding the cocoa bean.

Among the most important changes that cocoa undergoes during its fermentation, is the degradation of polyphenols (epicatechin, catechin, procyanidin, cyanidin and leucocyanidin, among others), which play an active role in the change of color, and its bitter, astringent taste^11,12^. Reduction of polyphenols takes place when they encounter polyphenol oxidases and peroxidases, enzymes responsible for catalyzing oxidation of polyphenols and formation of tannins—insoluble compounds with high molecular weight, also associated with astringent flavors in cocoa^13,14^. All degradation reactions are favored by high temperatures and long exposure times.

Proteins and amino acids also play a determinant role in the sensory quality of cocoa by being responsible for generating cocoa’s aromas and flavor precursors. Voigt et al.^15^ and Biehl et al.^16^ propose the catabolism of proteins, mediated by the enzymatic action of aspartate-endoproteases and carboxypeptidases, as the factor responsible for the release of hydrophobic amino acids and hydrophilic oligopeptides associated with the generation of aromatic notes, and interacting subsequently with reducing sugars during cocoa roasting.

In accordance with the above paragraph, the decisive role that enzymes play in developing the final quality of cocoa is evident. Therefore, controlling variables such as temperature and pH will result in a better control of the process and therefore better quality and homogeneity of the product, since these two factors are those that have the greatest effect on enzyme activity. However, there are other factors that affect enzyme activity. Aspartate-endoproteases decrease with the degree of maturity of the cocoa bean, while the concentration of peroxidase increases with maturity. Thus, the state of maturity also affects the final quality of the cocoa^15^. The optimum working pH of the enzyme’s aspartate proteases is 3.5 and 5.5 and of the carboxypeptidases is 5.8, yet since the two enzymes must act together, the optimum pH for the release of these aroma precursors has been found to be between 5.0 and 5.5^17^. In this pH range, polypeptides, corresponding to globulin 7S or vicillin are selectively degraded subunits^15,18^. Therefore, suitable conditions for the release of these aroma precursors involve a combined action of aspartate-endoproteases and carboxypeptidases, a pH between 5.0 and 5.5 and the presence of 7S globulins or vicillins. The maximum activity of peroxidase and polyphenol oxidase is a pH between 5 and 5.5 for the former and a pH of 7.0 for the latter.

Recent studies show that dietary polyphenols are effective antioxidants and play a significant role in the prevention of degenerative diseases^19^ (Manach et al.^22^). Consequently, these polyphenols can be considered part of the future of prevention and therapy in the area of medicine^20^. Cocoa polyphenols are associated with having beneficial effects on cardiovascular and inflammatory diseases, metabolic disorders, and cancer prevention^3,21^. Manach et al.^22^, Rodríguez-Carrasco et al.^3^ draw attention to the origin of the cocoa and manufacturing process, which affect the polyphenols’ content.

The objective of this research was to follow up on the biochemical and physicochemical changes that cocoa undergoes during its in-situ fermentation process, in three contrasting producing regions of the country, to test the hypothesis that monitoring the pH and temperature during fermentation can be an indicator of the quality of the cocoa obtained, regardless of the effect of the materials to be fermented and the production conditions.

## Results

### Fermentation system

**Figure 1:**
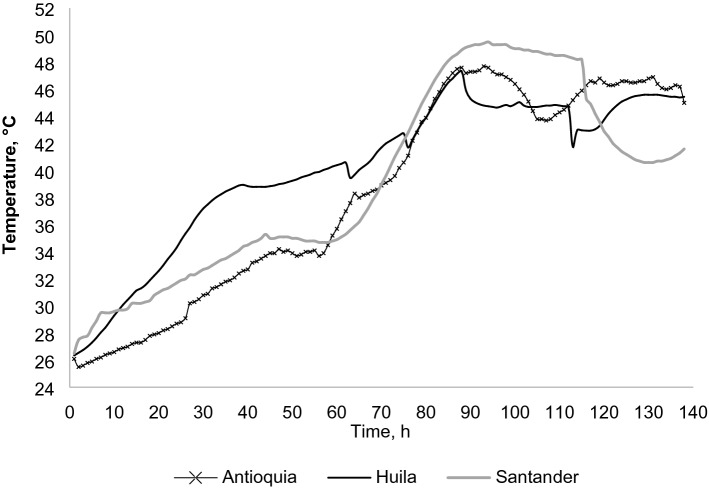
Temperature profile in the fermenter at the respective locations in the departments of Antioquia, Huila, and Santander.

Figure 1 illustrates the profile of temperatures measured in the center of the fermenter at each location. Ambient temperature and temperature on the surface of the fermenter were also recorded. Observation of the three temperatures showed that when the ambient temperature decreased, the difference between the temperature on the surface and the temperature in the center of the fermenter became greater, even if the fermenters were covered and located in closed spaces.

Results showed that Huila had the highest average environmental temperature, registering a value of 26.01 °C, followed by Santander (25.38 °C) and Antioquia (22.71 °C). The average temperature of the fermenter throughout the process was 39.89 °C in Huila, 39.43 °C in Santander and 36.86 °C in Antioquia.

As shown in Fig. 1, temperature changes in Antioquia and Santander were slower, while in Huila temperature increased significantly in the first hours of fermentation. Nonetheless, the highest fermentation temperatures were attained in Santander and Antioquia.

### Physicochemical properties

#### Shell moisture

**Figure 2:**
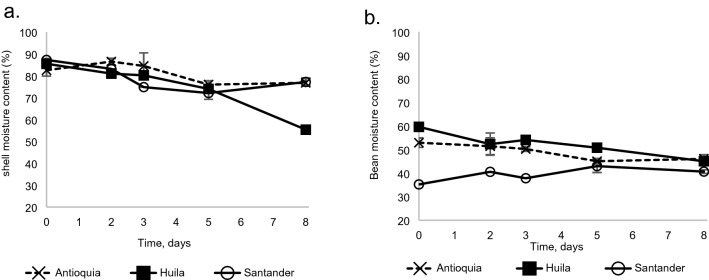
Moisture content of cocoa during fermentation carried out at three different locations: Antioquia, Huila, and Santander (**a**) shell and (**b**) beans.

The locality factor showed no effect on moisture content of the cocoa bean shell. However, on the fifth day of fermentation, humidity was reduced (p < 0.05) by 9%, 16% and 21% for Antioquia, Huila and Santander, respectively (Fig. 2a).

#### Cocoa bean moisture

Cocoa beans from Santander showed significant differences in moisture content, compared to those from Huila and Antioquia (p < 0.001). After 120 h of fermentation, moisture content decreased significantly (p < 0.01) by 17% in Antioquia and Huila, while increasing 18% in Santander (Fig. 2b).

#### TSS

Total soluble solids in the pulp did not show significant differences among localities or during fermentation, despite a reduction between 50 and 70% during the first two days of fermentation, remaining constant until the end of fermentation, with values between 4.5 and 6.5% for the three localities. A non-significant increase in TSS was observed in the beans, reaching values of 1.3% ± 0.1.

#### Acidity of the pulp

**Figure 3:**
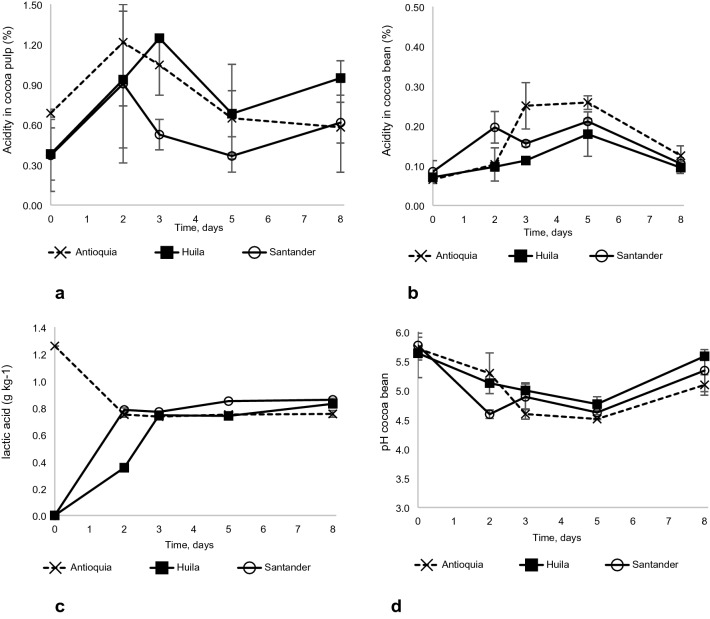
Dynamics of four variables during cocoa fermentation in three locations; Antioquia, Huila and Santander: (**a**) Acidity of the pulp, (**b**) acidity of the bean, (**c**) concentration of lactic acid, and (**d**) pH of the bean.

This variable did not report significant differences due to location or its dynamics; however, a peak in acidity was observed on the second day of fermentation for Antioquia and Santander, and on the third day for Huila, and then fell until the end of fermentation (Fig. 3a).

#### Acidity of the bean

No significant differences were found among sites. Acidity of beans increased significantly in Santander during the first two days of fermentation, while in Antioquia this increase took place on the third day. In the three localities the highest bean acidity was reached on the fifth day, with values of 0.26% in Antioquia, 0.21% in Santander and 0.18% in Huila (Fig. 3b).

#### Citric acid

There were no significant differences, although the three locations showed different behavior without a clear trend.

#### Lactic acid

The greatest significant differences, both in terms of location and fermentation, were found for lactic acid content. Nonetheless, after the first three days, the differences disappeared. In Antioquia initial lactic acid content was high (1.26%), on the second day it decreased to 0.75% (p < 0.05) and increased slightly to 0.76% at the end of fermentation. In the case of Santander and Huila, lactic acid content increased significantly until days 2 and 3 (0.78% and 0.75%, respectively), with values of 0.83% for Huila and 0.86% for Santander at the end of the fermentation process.

#### Acetic acid

There were differences in the dynamics, in which Santander presented a significant increase (1.83%) (p < 0.001) on the second day; while in Antioquia and Huila the significant changes (p < 0.05) were reported on the third day (Fig. 3c).

#### pH

During fermentation, pH decreased significantly (p < 0.001) starting on the second day in Santander and on the third day in Antioquia (p < 0.01) and Huila (p < 0.05). At the end of fermentation, pH reached values of 5.1, 5.6 and 5.4, for Antioquia, Huila and Santander (Fig. 3d).

#### Ashes

Ash content was around 2.2 ± 0.2, decreasing by 15%, 8% and 23% in Antioquia, Huila and Santander, respectively. Differences were significant only for Santander and Huila (p < 0.05).

#### Minerals

Minerals found in the highest concentrations in cocoa were zinc (46%), iron (26%), copper (24%), manganese (22%), and boron (11%). The iron, copper, and zinc contents were significantly higher (p < 0.05) for cocoa from Huila. During fermentation, iron, manganese, boron and zinc decreased by 32%, 19%, 17% and 12%, respectively in Huila; while in Antioquia, iron, boron and zinc decreased by 12%, 9% and 7%. Santander, on the other hand, reported an increase in iron (16%) and boron (33%). Potassium, phosphorus, magnesium and sulphur were found in smaller quantities in percentages of 0.83%, 0.41%, 0.24% and 0.12% respectively.

#### Fats

The content of fats in the cocoa was not significantly different among localities or in response to the fermentation process, with values of 53% for Santander and Antioquia, and of 49% for Huila.

#### Fatty acid profile

These profiles did not present significant differences. The highest contents of fatty acids in the three locations were represented by palmitic (28.5%), oleic (34.5%) and stearic (33%) acids and their changes during fermentation were less than 5%.

#### Protein

The protein content of cocoa from Huila was significantly higher than that from Antioquia. In the three locations protein content showed a significant decrease (p < 0.05) of 12% for Antioquia and Huila, and 16% for Santander, reaching values of 12.5 ± 0.3% for the three locations.

### Biochemical properties

Amino acids, sugars, polyphenols, methylxanthines have been included in this group, given their role in the quality of cocoa.

#### Total sugars (TS), reducing sugars (RS)

**Figure 4:**
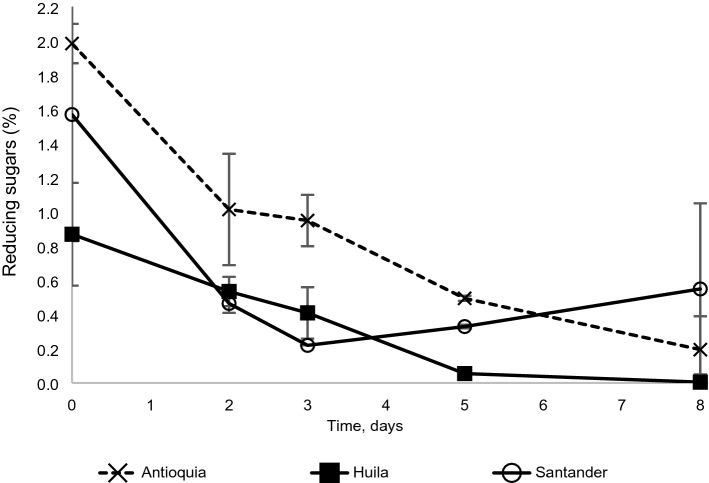
Dynamics of the content of reducing sugars during cocoa fermentation in three contrasting locations.

No significant differences were found in TS content in raw cocoa from Antioquia (5%), Huila (2.8%) and Santander (4%), however at the end of the fifth day of fermentation, these values were reduced by 55, 64 and 57%, respectively. RS content in fresh beans from Antioquia (1.99%) was significantly higher (p < 0.05) than those from Huila. During fermentation, the RS decreased (p < 0.01) in those cocoa beans for Antioquia (75%) and Santander (79%). Although the decrease for Huila was 94%, it was not significant (Fig. 4).

#### Amino acids

Lysine (3.4%), phenylalanine (1.9%), alanine (2.5%), valine (1.8%), serine (1.6%), glutamine (1.4%), proline (1.3%), leucine (1.1%) were the amino acids present in greater proportions. Others like isoleucine (1.07%), tyrosine (0.75%), threonine (0.43%), glycine (0.13%), histidine (0.05%) and arginine (0.045) were found in lower proportions. Despite the low concentration they presented, the location and the fermentation process caused significant differences in the amino acids profile. In Antioquia and Huila, amino acids content decreased significantly by about 11% on the fifth day, while in Santander, the reduction was higher, 14% for most of the amino acids present.

#### Total polyphenols (TP)

**Figure 5:**
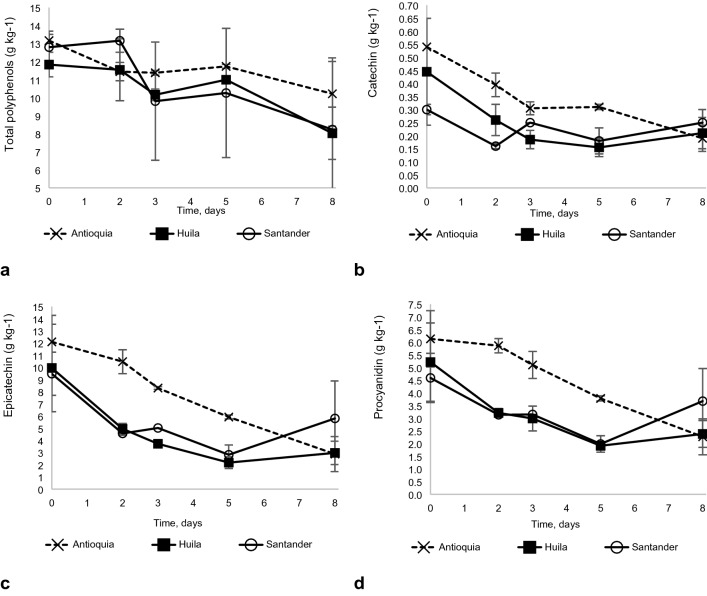
Dynamics of the polyphenols throughout the cocoa fermentation process in three locations: Antioquia, Huila, and Santander. (**a**) Total polyphenols, (**b**) Catechin, (**c**) Epicatechin and (**d**) Procyanidin.

Although the polyphenols content decreased 11%, 7% and 20% during the fermentation in each of the three locations Antioquia, Huila and Santander, respectively, there were no significant differences (Fig. 5a).

#### Catechin

This flavanol was reduced by 43%, 65% and 40% in Antioquia, Huila and Santander, respectively, due to the fermentation process. Nonetheless, only in Huila, was the reduction significant (p < 0.05) (Fig. 5b).

#### Epicathequin

This polyphenol, although present in a higher proportion in fresh beans, was reduced by 51%, 78% and 71% in Antioquia, Huila and Santander, respectively, on the fifth day of fermentation (Fig. 5c).

#### Procyanidins

This flavonoid was decreased by 38%, 53% and 67% in Antioquia, Huila and Santander, during the fermentation (Fig. 5d). The reduction was significant (p < 0.05) for the three locations.

#### Theobromine

No significant differences were identified, for either of the factors. The theobromine content in fresh beans was between 9.5 and 11 g kg^−1^ and at the end of the fermentation, decreased to values around 8.7 g kg^−1^ in the three locations.

#### Caffeine

The content of this methylxanthine in fresh beans was higher (p < 0.05) in Antioquia, (3.19 g kg^−1^). After the fifth day of fermentation the reduction in caffeine content was 33, 28 and 6% for Antioquia, Huila and Santander, respectively, reaching values around 2.19 g kg^−1^ in the three locations.

#### Theobromine/caffeine, T/C

This correlation showed significant differences (P < 0.05) between fresh beans from Antioquia and Huila; yet aforementioned differences disappeared during the fermentation process. At the end of the process this T/C ratio reached values between 4.5 and 4.8.

#### Antioxidant capacity

The antioxidant capacity did not present a clear behavior in the three locations. At the end of fermentation, the antioxidant capacity of the fermented cocoa beans decreased for Huila, but increased for Antioquia and Santander.

### Aroma profile

This analysis was aimed at identifying compounds associated with some of the most recognized and valued aromas and flavors in the fine cocoa market; as well as the factors responsible for reducing its quality, such as acetic acid. In previous studies, 2,3-butanediol acetate, 2-phenyl ethanoate and ethyl dodecanoate were identified as the aromatic compounds prevailing in cocoa liquors coming from these localities (data not shown), with concentrations between 15 and 30 mg L^−1^ and conferring fruit notes to cocoa. The present study is focused on identifying other compounds that could make important differences. However, none were identified that exceeded the concentration of those previously found. Benzaldehyde and ethylbenzoate content decreased during fermentation, while 2,3,5-Trimethylpyrazine, Linalool oxide, and phenethyl alcohol increased. Nonetheless, none of these changes were significant. The compounds identified in higher concentration in cocoa from the different locations were acetic acid, 2,3-butanediol, linalool oxide, phenethyl alcohol, 2,3,4,5-tetramethylpyrazone. These compounds also maintained high concentrations in the cocoa liquor, except for 2,3,4,5-tetramethylpyrazone, which decreased significantly (p < 0. 01); while 2,3,5-Trimethylpyrazine increased significantly (p < 0.01). In Huila, three compounds were outstanding: 2.5-Dimethylpyrazine with concentrations of 11.1 ± 0.14. Mg L^−1^; linalool oxide (5.85 mg L^−1^ ± 1.0) and phenethyl alcohol (7.5 ± 4.8 mg L^−1^). In Santander, the most prevalent compounds were phenethyl alcohol (8.05 ± 2.3 mg L^−1^), 2,3-dimethylpyrazine (3.25 ± 1.34 mg L^−1^) and 2.3-butanediol (2.45 ± 0.78 mg L^−1^). In Antioquia, the main compounds were phenethyl alcohol (6.3 ± 4.8 mg L^−1^). 2,3,4,5-tetramethylpyrazone (5.4 ± 3.8 mg L^−1^) and 2,3-butanediol (4.1 ± 2.4 mg L^−1^). Other compounds identified were phenyl-acetaldehyde, which was present in a greater proportion in cocoa from Antioquia (p < 0.05), but the content in liquor was significantly less (p < 0.05); acetophenone was present in greater concentration in cocoa than in liquor in the localities of Antioquia and Huila (p < 0.01); 2-Acetyl pyrrole was present in liquor from Huila in greater proportions than in liquor from other locations (p < 0.05).

### Sensory profile

**Figure 6:**
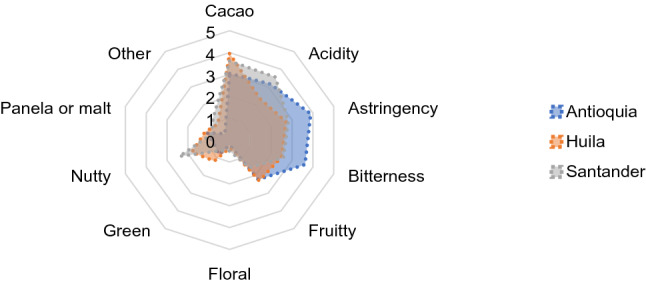
Sensory profile of cocoa liquor from Antioquia, Huila and Santander*.*

The Kruskal–Wallis test for the parameters evaluated by sensory analysis showed that the liquor from Huila had significantly higher cocoa (P < 0.02) and nut (P ≤ 0.01) aromas; and lower astringency (P ≤ 0.01), acidity (P ≤ 0.01), and bitterness (P ≤ 0.01) than that from Antioquia. Cocoa produced in Santander had a remarkably similar profile to that of Huila, although it was significantly (P ≤ 0.01) less acidic. As for the floral, fruit, green and panela or malt aromas, there were no significant differences among the liquors of the locations under study (Fig. 6).

Through Spearman’s correlation analysis, a direct and significant relationship (P < 0.02) was found among nut, fruit, and cocoa aromas (Table 1). In contrast, off-flavors associated with astringent and bitter profiles were inversely correlated with the nutty aroma. Acid flavors were directly associated with the over-fermented profile (other) and inversely with the unripe notes (Table 1).

**Table 1:** Spearman correlation among sensory variables.

Correlation matrix among sensory variables										
Flavors	1	0.003	** − 0.260**	**− **0.166	0.118	0.062	0.057	**0.253**	0.121	0.129
Cacao	0.003	1	0.014	0.225	0.192	0.213	**− 0.376**	0.208	0.041	**0.325**
Acidity	**− 0.260**	0.014	1	**0.413**	**− **0.012	0.085	0.067	**− 0.365**	0.014	**− **0.059
Astringency	**− **0.166	0.225	**0.413**	1	0**.**085	**− **0.033	**− **0.077	**− 0.357**	**− **0.052	0.073
Bitterness	0.118	0.192	**− **0.012	0.085	1	0.210	0.117	**0.260**	0.150	0.107
Fruity	0.062	0.213	0.085	**− **0.033	0.210	1	0.171	0.144	**0.279**	**− **0.011
Floral	0.057	**− 0.376**	0.067	**− **0.077	0.117	0.171	1	**− **0.041	0.065	**− **0.229
Green	**0.253**	0.208	**− 0.365**	**− 0.357**	**0.260**	0.144	**− **0.041	1	0.105	**0.256**
Nutty	0.121	0.041	0.014	**− **0.052	0.150	**0.279**	0.065	0.105	1	**− **0.079
Malt	0.129	**0.325**	**− **0.059	0.073	0.107	**− **0.011	**− **0.229	**0.256**	**− **0.079	1
Other	1	0.003	**− 0.260**	**− **0.166	0.118	0.062	0.057	**0.253**	0.121	0.129

In bold, significant values (P < 0.05). Other: refers mainly to the over-fermented profile.

## Discussion

### System conditions

In Antioquia, where the average temperature is lower than in Huila and Santander, the dynamics and the characteristics of aromatic and sensorial quality of liquor obtained through the fermentation process presented important differences. Although the increase in temperature is due to exothermic microbiological reactions that are generated during the fermentation of the beans, at lower ambient temperatures, heat loss from the fermenter is higher, reducing the temperature inside the fermenter. This brings consequences on the generation of flavor and aroma precursors, which then result in both a poor aromatic and sensory profile of the cocoa. Although the role of relative humidity is not so clearly defined, when this condition is very high and ventilation scarce, fungi development is favored and off-flavors are generated.

### Physicochemical properties

#### Physicochemical changes in the pulp

When fermentation starts, the pulp is highly viscous, a condition that limits the entry of oxygen into the system, creating an anaerobic environment, which—added to the high content of sugars and organic acids—promotes the multiplication of yeasts^23–25^. Yeasts use sugars as a substrate, releasing different metabolites, such as ethanol, and energy that increases the temperature of the system, as shown in Fig. 1. The scarcity of oxygen, together with the presence of citric acid and residual sugars, favors the development of lactic bacteria^26^. These factors explain the rapid decrease in TSS found in the first 48 to 72 h of fermentation and the increase in temperature, between 38 and 45 °C. Additionally, the yeasts release pectinolytic enzymes, which break down the walls of the plant cells, increasing the fluidity of the pulp^5,27,28^). This greater fluidity, on the one hand, causes the pulp to drain out of the system, and with it, sugars, acids, and moisture—which was confirmed with the results obtained from the analysis of the pulp. On the other hand, the greater fluidness also favors the entry of oxygen into the system generating aerobic conditions, which, together with the reduction of acidity after the second day and the increase in temperature, favored the development of acidic-acetic bacteria^26,29^—which in turn explains the presence and increase of different metabolites, among them acetic acid that increased until day 3 and 5 of fermentation. The results showed no significant differences among sites, although Huila reported a significant increase in acidity until the third day and, despite the drastic decrease suffered until day 5, it ended up with the highest content.

#### Physicochemical changes in the bean

##### Moisture and sugars

The cocoa beans lost humidity during fermentation, initially due to the diffusion of moisture towards the outside of the grain, and later during the turning process, favored by the low relative humidity of the atmosphere. However, moisture content was high enough—at least 35%, according to Apriyanto et al.^30^—to favor the enzymatic processes that develop inside the almond. Sugar content ranged between 1.9 and 3.5%, values that are within the 0.39% to 3.48% range defined by Peláez et al.^31^, Graziani de Fariñas et al.^32^, Misnawi et al.^23^. Reducing sugars can decrease throughout the fermentation by their participation as a source of energy in the different biochemical reactions and by the enzymatic breakdown of carbohydrates, releasing the respective mono-, di- and oligosaccharides^33^. On the other hand, reducing sugars can also increase during fermentation due to the hydrolysis of sugars caused by the acidification of the bean and the diffusion of reducing sugars from the pulp. Then, the final change in RS content is determined by the global balance among these two types of reactions.

##### Acidity and pH

The results regarding acidity are in line with results found for lactic and acetic acid, and with what was proposed by De Vuyst and Weckx^33^ and Wacher^26^. Citric acid did not show significant changes, while lactic and acetic acids increased their concentration from the second to the fifth day of fermentation. These acids diffuse from the pulp to the bean and together with the increase in temperature, cause the death of the embryo, triggering a series of biochemical reactions^28,34,35^, which begin with rupture of the membranes of the internal compartments, favoring the mixing of their contents and the activation of enzymes. Enzyme activity is affected by both the temperature and the pH of the environment. Fermentation carried out in Huila presented a less drastic reduction of pH throughout the process, reaching minimum values of 4.8, compared to 4.6 and 4.5 presented by Santander and Antioquia, respectively; pH values later increased to values of 5.6 (Huila), 5.3 (Santander) and 5.1 (Antioquia). These small differences in pH can however make a significant difference in the final quality of the cocoa. According to Jackels and Jackels^36^, if pH of the beans reaches a range between 5.5 and 5.8 during the process of acidification, fermentation is considered to be incomplete,if pH is less than 4.75 the batch is considered to be over-fermented or very acidic; while a pH between 4.75 and 5.19 indicates a good fermentation process. Afoakwa et al.^37^ report that the generation of better aromatic and sensory profiles in cocoa is achieved when the pH is maintained between 5 and 5.2 and the temperature is kept below 40° C. Biehl et al.^38^ affirm that under these conditions, the action of the aspartate endoproteases and carboxypeptidases enzymes is more efficient and that they achieve a selective release and breakage of proteins, which play a decisive role in the generation of aroma and flavor precursors. Fermentation in Huila was the closest to the favorable conditions specified by the above authors and which was corroborated by the biochemical reactions and the sensory profile of samples from this site.

##### Ashes and minerals

Values for ash content were below the 3% reported by Afoakwa et al.^5^ but its dynamic corresponded to those indicated by other authors^5,39^. This reduction can be explained by the ashes leaching with the mucilage that the system leaves, since most of the minerals are in the cocoa bean shell, which facilitates the exit of minerals from the system, especially the soluble ones^40^.

The acid soils from Huila, Table 2., allowed higher solubility of minerals such as Fe, Cu, Mn, which was reflected in the higher presence of these minerals in fresh cocoa beans from Huila (45, 33, 22 mg Kg^−1^ respectively), than those from Antioquia (24, 13 and 17 mg Kg^−1^) and Santander (15, 8 and 10 mg Kg^−1^) respectively.

**Table 2:** Edaphoclimatic characteristics of the three study locations.

Edaphoclimatic characteristics	San Vicente de Chucuri (Santander)	Maceo (Antioquia)	Rivera (Huila)
Average annual temperature	12– > 28 °C	20–26 °C	16–28 °C
Average total annual rainfall	1500–3000 mm	2500–3000 mm	1000–2000 mm
Potential monthly evapotranspiration	1400–1600 mm	1400–1600 mm	1200–1600 mm
Relative humidity	80–85%	80–85%	70–75%
Thornthwaite water index	Humid	Humid	Subhumid humid
Pluviometric Regime	Bimodal 2	Bimodal 2	Bimodal 6
Average soil moisture (Thornthwaite water index)	Humid (80 > = hi > 60)	Humid (80 > = hi > 60)	Subhumid humid (20 > = hi > 0)
Soils	Troporthents, Dystropets, and Eutopepts. Deep to shallow soils with organic matter adequate content, medium natural fertility, topography strongly sloping to steep (25%, 50% and up to 100%), medium to high susceptibility to erosion	Dystropepts and Troporthent, medium to very deep soils, low natural fertility, undulating to severely broken topography (12–75% slopes), and high erosion susceptibility	Troporthents, Ustorthents, Dystropepts. Medium to shallow soil depth; from clayey textures and sandy in granite areas; stony in the profile, medium to low content of organic matter, pH from 4.5 to 5.0; strongly undulating topography (25%—60% slope), alternating with abrupt sectors (100% slope), susceptibility to high to medium erosion
Rainfall Pattern	Bimodal 2: Rainy season from March to May and September to November The mid-year dry season is not very marked	Bimodal 2: Rainy season from March to May and September to November The mid-year dry season is not very marked	Bimodal 6: Two rainy seasons at the beginning and the middle of the year; Dry season between June and August is much more deficient than that of the first quarter

Source: IDEAM^56^, Gómez et al.^57^.

In terms of minerals, phosphorus content was found to be similar to that reported in cocoa from Ecuador^41^; and about double the content reported in cocoa from Ghana^5,41^. The calcium and potassium values were lower than those reported by Afoakwa et al.^5^ for Ghanaian cocoa—0.14% and 2.3%, respectively. However, reports by Torres et al.^41^ for cocoa from Ecuador and Ghana show values of 0.12% and 0.11 for calcium and 1.25% and 1.2% for potassium, respectively. The values are closer to those found in this study. Micronutrients such as zinc and copper presented values close to those reported for cocoa from Ecuador and Ghana (45 and 37 mg kg^−1^ for zinc, and 26 and 24 mg kg^−1^ for copper, respectively). Manganese content was low compared to that reported by Torres et al.^41^, who found values of 21 and 39 mg kg^−1^ in cocoa from Ecuador and Ghana, respectively. Mineral content in cocoa depends largely on the site’s edaphic and climatic conditions, but there is no further information in the literature on the influence of these on the fermentation process, although it is known that the Ca, for example, can play an important role in different biochemical reactions in plants. The presence of sodium, calcium, potassium, magnesium, and iron salts, among others, can affect color; other elements such as magnesium, zinc, and copper can act as co-factors in enzymatic processes^42–44^.

##### Fats and fatty acids

This research found fat contents between 49 and 54%, which are within those reported in the literature, although values below 52% are considered to be low. Normal fat content lies between 52 and 55% and contents higher than 55% are considered high^45^.

Palmitic, oleic and stearic acids contribute with more than 90% to fatty acid content, but no significant differences were found among sites in terms of fatty acid content. These contribute to the development of the cocoa’s aromatic profile, but large amounts (> 1.75%) of fatty acids result in bland consistencies and greater susceptibility to deterioration.

### Biochemical properties

#### Proteins and amino acids

Fresh cocoa beans from the three locations presented protein content among 13% and 14%, lower than those (16% and 22%) reported by Afoakwa et al.^37^, yet in accordance with those values (10% to 15%) stated by Marseglia et al.^46^. Proteins from cocoa beans are composed basically of globulins and albumins. During fermentation, protein content dropped to values between 11.5 and 13.9%, which can be due to the vicilin (globulins) storage protein degradation by the action of an aspartic endoprotease and a serine carboxy(Exo) peptidase, which release hydrophilic peptides and hydrophobic amino acids^15^ (De Vuyst and Weck^33^). The increase of hydrophobic amino acids such as leucine, alanine, phenylalanine observed in the present study, confirmed this proposal.

#### Total polyphenols

Polyphenols in fresh cocoa beans ranged from 11 to 13% for the three locations. These values are significantly higher than those reported by Miller et al.^47^ and Perea et al.^48^ of 1.3% and 6%, respectively. The higher content found in the present study could be due to the type of material and its degree of maturity, among other factors^49^. However, during the fermentation process, the polyphenols decreased due to their diffusion from the storage cells and further oxidation by the action of polyphenol oxidase and the peroxidase released by the breakage of cell membranes inside the beans, which promotes the generation of highly insoluble tannins of high molecular weight^13,14^. The extension of these changes depends on two factors, the enzyme concentration and the advance of the fermentation. Peroxidase concentration and activity increase with the fruit ripeness and during the fermentation increase until the third day, after which decrease.

Cocoa polyphenols are mainly composed of proanthocyanidins (58%-65%), catechins (29–38%) and anthocyanins (4%). Epicatechins, procyanidins and catechins are polyphenols that play a decisive role in cacao quality and bioactivity. Although epicatechin was present in high amounts in fresh cacao beans, the fermentation decreased it between 51 and 78% in all three locations. Catechin and procyanidin declined between 40 and 63%, which is in line with the study by Nazarudin et al.^10^. The high reactivity and selectivity of peroxidase for epicatechin explain the higher reduction in epicatechin. The change of epicatechin content is a progress fermentation indicator and its reduction contributes to less astringent cocoa products, favouring its quality^49^.

#### Antioxidant capacity

Although polyphenols are known as antioxidants, and procyanidins are often directly correlated with antioxidant capacity^49–51^, in this study, the epicatechin, catechin and procyanidins decreased significantly during fermentation, but the antioxidant capacity remained stable. Thus, antioxidant capacity is affected by other types of polyphenols or compounds not considered in this study.

#### Methylxanthines

Theobromine and caffeine constitute 99% of the alkaloid content in cocoa; the rest corresponds to theophylline and salsolinol. These compounds are associated with the bitter taste of cocoa. Contents of theobromine (8.4 to 11.4 g Kg^−1^) and caffeine (1.4 to 3.2 g kg^−1^) found in the present study were lower than those reported by other studies: 18 to 28 g kg^−1^ for theobromine and 3 to 10 g kg^−1^ for caffeine^45^. Results on the evolution of theobromine during fermentation agree with findings of Brunetto et al.^52^ who proposed that theobromine increases during the first hours of fermentation and decreases after 72 h^6,7^. Davrieux et al.^53^ explain this initial increase by the fact that in the first days the mucilage contains theobromine that diffuses into the inner part of the grain. On the other hand, Krähmer et al.^2^, Palacios^54^ and Aprotosoaie et al.^6,7^ propose that the decreasing of this compound is due to the exudation of the beans during fermentation, favored by the increase in temperature. Concentrations of both caffeine and theobromine were reduced during fermentation. Theobromine/caffeine ratio depends on the genetic material, the state of maturity and the growing conditions^6,7,53^. The 3:7 theobromine/caffeine ratio found in fermentations in this study points out that raw cacao corresponds to Trinitarian cocoa materials.

#### Aromatic profile

Free amino acids, short-chain peptides and reducing sugars released during fermentation play a crucial role in the Maillard reactions during the roasting stage, generating mainly aldehydes and pyrazines, that along with esters, alcohols and ketones comprise more than 600 volatile compounds that confer cocoa, nutty, malt, fruity and flowery notes to cacao products. However, some acids and phenols can cause undesirable notes. The fermentation generated aroma precursors expressed during roasting and reduction of compounds like 2,3,4,5-tetramethyl pyrazine during the fermentation process can cause this to occur. This pyrazine showed higher contents in fresh cocoa beans than in cocoa liquor. Cocoa liquor from Huila presented 2,5-dimethyl pyrazine, linalool oxide and phenethyl alcohol, which conferred cocoa, floral and fruity notes to the cocoa liquor. However, cocoa liquor from Huila was also highly acidic, associated with the presence of acetic acid. In the case of Antioquia and Santander, fresh cocoa beans showed a high content of acetic acid and tetramethylpyrazine. During fermentation in Antioquia and Santander, acetic acid diminished by 74% and 67%, and tetramethylpyrazine by 66% and 94% respectively. Meanwhile, other compounds such as dimethyl and trimethyl pyrazine, linalool oxide and phenethyl alcohol increased during fermentation.

#### Sensory analysis

Cocoa liquor is the paste derived from cocoa beans, and Fig. 6 depicts its aroma and flavour intensity. The cocoa liquor from Huila had a better sensory profile than that of Antioquia and Santander, evidenced in its powerful cocoa aroma, nutty notes, less astringent sensation, and neither sour nor bitter taste. The fermentation of the cocoa generates a 40% reduction in the content of initial polyphenols associated with the level of bitterness and astringency in the cocoa liquor, which allows the expression of specific aromas and explains the direct and significant relationship between the nutty, fruity, and cocoa aromas, found in Spearman's correlation analysis, and that can be an indicator of efficient fermentation processes.

The sensory profile is the most representative indicator of cocoa quality since each parameter evaluated (texture, colour, aroma, flavour) consolidates the effects of all changes occurring from production to processing. The study confirmed cocoa beans from Huila to be the best quality and also the best fermentation performance supported in (1) the reduction of epicatechin, catechin and procyanidins that led to a reduced sensation of astringency and bitter taste, and appropriate colour; (2) the degradation of proteins and the release of hydrophobic amino acids and hydrophilic oligopeptides that led to a better generation of aroma and flavour precursors. All these changes are reflected in the cocoa liquor, after drying and roasting.

These excellent profiles shown by the cocoa process from Huila, are explained by a better development in the dynamics of pH and temperature shown during cocoa fermentation, and whose effect is decisive on the biochemical reactions that occur inside the bean and on the dynamics of fermentation. Temperature and pH strongly affect the activity of the enzymes that regulate the crucial biochemical changes inside the bean, such as the oxidation of polyphenols and the catabolism of proteins. Temperature and pH of the medium largely determine the sequence of microbiological groups and metabolites responsible for triggering changes within the grain during fermentation.

The fruity and floral notes present in the liquors from the three departments are associated with the presence of esters and alcohols, which are stable to the processes of transformation of the grain^55^, and qualify them as both fine quality and of aroma.

## Conclusions

The study of the cocoa fermentation process in the three different production areas in Colombia showed that there are two stages involved in the final quality of the bean: the first in which the sugars and acids are fermented to generate the metabolites and second, the conditions that cause the death of the embryo and trigger a whole series of reactions inside the bean. These biochemical reactions are responsible for the generation of aroma and flavor precursors, which will later build the final quality of cocoa and will be consolidated in the sensory and aromatic profile of the same. In these two stages, both the temperature and pH profiles of the system play a determining role. In fact, they should be the principal variables to be monitored and controlled. These two variables have a direct effect both on the biochemical reactions inside the grain, thanks to their effect on the activity of the respective enzymes and on the development of fermentation—by affecting both the sequence of microorganisms that take part in the fermentation process and the metabolites they release, and the changes that this trigger inside the grain.

## Materials and methods

### Location

This research was carried out in the three most important cocoa-producing agroecological zones in Colombia: (a) municipality of San Vicente de Chucurí (coordinates: 6° 52″57″N; 73° 24″ 46″ O) in the mountains of the department of Santander, (b) municipality of Rivera (coordinates: 2°46′38″ N; 75°15′19″ O) in the inter-Andean valleys of the department of Huila, and (c) municipality of Maceo (coordinates: 6°33′12″N 7; 4°47′14″O) in the marginal coffee zone in the department of Antioquia. The edaphoclimatic characteristics of each zone are described in Table 2. The purpose was to compare and determine the effect of edaphoclimatic conditions and genetic material on the final quality of cocoa. To achieve this objective, a representative farm was selected in each agroecological zone, where a great diversity of hybrids and clones were found in each region. Considering that the three sites (Maceo, San Vicente de Chucurí and Rivera) are representative of Colombian cocoa production, this document will refer to the departments where they are located: Antioquia, Santander and Huila, respectively. The objective of extrapolating from site to department is estimating the quality of cocoa obtained by the interaction with the environment at the scale of cocoa growing regions in Colombia.

Cocoa beans embody the raw material to produce cocoa-based products. Cocoa beans are the seeds of the fruits of the cocoa tree (Theobroma cacao L.) and consist of two cotyledons or nibs and an embryo, all enclosed in a shell. They are built for storage cells contained proteins, starch, and fat globules; and for pigmented cells, which comprise polyphenols and methylxanthines.

### Monitoring of fermentation conditions

Both fermentation room and fermenter conditions were monitored.

#### Temperature (T) and relative humidity (RH)

ThermaData® HTD (Worthing, United Kingdom) humidity and temperature loggers were placed in the fermentation rooms to monitor T and RH. The logger was hung 0.30 m from the centre of the roof, far from any surface. This information is relevant to understand how environmental conditions can affect fermentation performance. Low external temperature can generate a loss of heat from the fermenter system to the environment affecting the fermentation performance. Loggers registered the T and RH from the beginning until the end of the process, every 20 min.

#### Fermentation temperature

To get more accurate information about the fermentation process, the profile temperature along the fermentation process was recorded using a Testo 560 7351 (Testo SE & Co, Lenzkirch, Germany). Thermocouples installed in the fermenter registered the temperature of cocoa grains under fermentation every 20 min.

### Monitoring fermentation dynamics

#### Physicochemical analysis

##### Moisture of the shell and the bean

A slight modification of the AOAC Method 931.04 was used to determine moisture content. For this purpose, 20 beans were taken in each sampling from three different points of the fermenter. The testa or bean shell, to which the pulp is attached, was removed. Both shell and beans were placed in glass capsules and weighed separately. They were placed in a Memmert drying oven (Model VM400, Schwabach, Germany) at 103 °C for approximately 48 h, and were then transferred to the dryer at 12-h intervals and weighed until a constant weight was obtained.

##### Total soluble solids (°Brix)

AOAC 932.12^58^ protocols were followed to determine total soluble solids, expressed in ° Brix, using a digital refractometer (Atago PAL-1, Tokyo, Japan). One drop of the previously homogenized extract was placed on the equipment’s prism to record the value.

##### Sugars (reducing and non-reducing)

Sugars were determined by liquid chromatography following the methodology proposed by Murillo et al.^59^: 1 g of cocoa sample was dissolved in 10 mL of a 0.2% (w/v) benzoic acid solution and centrifuged at 5000 rpm for 30 min. Finally, the supernatant was filtered with a PDVF 0.22 μm syringe filter and injected into the Thermo Dionex Ultimate 3000 HPLC liquid chromatograph (Thermo Fisher Scientific Inc. Asheville, United States) equipped with degasser, automatic injector, an ICE COREGEL 107-H column (Transgenomic) and an RI RefractoMax520 detector, with an injection volume of 20 µL.

##### Titratable acidity of bean and pulp

The protocol established by AOAC 942.15^60^ was followed, using a Hanna potentiometer (HI2020-01, Woonsocket, Rhode Island, United States), to determine acidity of an acid–base titration of 2 ml of the previously diluted bean extract, which was titrated with NaOH (0.03 N) until a pH of 8.2 was obtained. The titratable acidity was quantified as the equivalent weight of citric acid.

##### Organic acids (citric, lactic and acetic)

1 g of cocoa was weighed and dissolved in HCl by adjusting the pH to 2.3. Finally, it was centrifuged and the supernatant was injected into a Thermo Dionex ultimate 3000 HPLC system (Thermo Fisher Scientific Inc. Asheville, United States) equipped with degasser, automatic injector, an ICE COREGEL 107-H column (Transgenomic) and an RI RefractoMax520 detector, with an injection volume of 20 µL and a flow rate of 0.6 mL/min.

##### pH

The AOAC 943.02^61^ potentiometric method was used to determine pH, with a Hanna (HI2020-01,Woonsocket, Rhode Island, United States) pH meter, previously calibrated, by introducing the equipment’s electrode directly in the juice extracted from the pulp and then recording the value.

##### Ashes

The official AOAC 942.05^62^ method was followed to determine ash content: 0, 1 g of the dried and ground sample was introduced into previously tared porcelain crucibles which were taken to a Thermo Scientific Thermolyne FD1540M-33 muffle (Thermo Fisher Scientific Inc., Asheville, United States), at 600 °C for 4 h. Once the flask had cooled, it was transferred to a desiccator until it reached room temperature for weighing.

##### Minerals

Atomic absorption spectrometry was used to determine mineral content of phosphorus (P), potassium (K), calcium (Ca), magnesium (Mg), sodium (Na), sulfur (S), iron (Fe), copper (Cu), manganese (Mn) and zinc (Zn): 0.5 g of the dried and ground sample was weighed and mineralized by the acid digestion method in a closed system (Milestone Ultrawave microwave) with nitric acid and hydrogen peroxide. One milliliter of the mineralization extract was added to a 50 ml beaker with 9 ml of a 0.040% lanthanum chloride solution to complete a final volume of 10 ml. The concentration of each mineral was determined by atomic absorption spectrometry, using calibration curves for each element.

##### Protein

The micro Kjeldahl method based on AOAC 960.52^63^ was used to measure crude protein content by determining the nitrogen content, taking 0.1 g of a dried and ground sample.

##### Fats

The Soxhlet gravimetric method proposed in AOAC 963.15^64^ was used to determine fat content, using petroleum ether for solvent extraction.

#### Biochemical analysis

##### Fatty acid profile

Following the methodology proposed in the ISO 5508:1990 norms, fatty acids were analyzed by obtaining and quantifying their methyl esters using gas chromatography with flame ionization detector (GC-FID). The chromatographic analysis of the sample was performed in a gas chromatograph (Agilent Technologies 7890 N, Santa Clara, United States), reference of the column used was HP-88 (J & W Scientific Folsom 60 m × 0.25 mm × 0.20 µm) and the injection was performed in split mode (10:1).

##### Amino acids

The cocoa sample was dissolved in 6 N HCl and placed in reflux for 22 h. It was then neutralized and brought to a pH of 2.3 with 0.1 N HCl. Subsequently, the amino acids were derivatized with o- phthalaldehyde and injected into the Dionex ultimate 3000 HPLC chromatograph (Thermo Fisher Scientific, Waltham, MA, United States) controlled by the Chromeleon 7.2 software, equipped with a reversed-phase column (Zorbax Eclipse XDB, measuring 150 mm × 2.1 mm) with a particle size of 5 μm.

##### Total polyphenols

Following the procedure described by Wollgast^65^, the Folin-Ciocalteu method was used to measure total polyphenols, using gallic acid as a reference standard to make the calibration curve and thus determine the concentration of gallic acid associated with the absorbance at 765 nm in a Thermo Scientific UV–VIS spectrophotometer (Genesys 10UV Thermo Electron, Waltham, MA, United States). Results were reported as gallic acid equivalents on a dry base.

##### Catechin, epicatechin, procyanidin (B1 + B2) and methylxanthines (caffeine and theobromine)

The methodology proposed by Carrillo et al.^66^ was followed, using the calibration curves of the respective reference substances. A Dionex ultimate 3000 HPLC system (Thermo Fisher Scientific, Waltham, MA United States) controlled by the Chromeleon 7.2 software was used for analysis of compounds. The system is equipped with a reversed-phase column (Zorbax Eclipse XDB measuring 150 mm × 2.1 mm) with a particle size of 5 μm. Theobromine and caffeine were quantified using a UV detector at 273 nm.

##### Antioxidant capacity

The DPPH (2,2-diphenyl-1-picrylhydrazine in 2,2-diphenyl-1-picrylhydrazine) assay reported by Sharma and Bhat^67^ was used to measure antioxidant capacity, deploying the antioxidant action of compounds containing –OH groups that discolor the DPPH reagent,this effect was evaluated by decreasing absorbance at 517 nm in a Thermo Scientific UV–VIS spectrophotometer (Genesys 10UV Thermo Electron, Waltham, MA, United States), using ascorbic acid as the reference pattern.

#### Aromatic profile

The HS-SPME technique by gas chromatography with flame ionization detector (GC-FID) was used to analyze volatile compounds. Linalool, 2,3-butanediol, 2,5-dimethylpyrazine, 2-phenethylacetate, acetophenone, 2,3,5-trimethylpyrazine, ethylbenzoate, phenethyl alcohol, acetyl pyrrole, tetramethylpyrazine, benzaldehyde, phenylacetaldehyde (Sigma-aldrich) and acetic acid (Merck) were used as reference standards. The analysis was performed on a gas chromatograph (GC) 7890 (Agilent Technologies, Santa Clara, United States) with a flame ionization detector (FID). The column used in the analysis was HP-5 (5%—polyphenol—95% dimethylsiloxane, 30 mx 0.32 mm × 0.25 µm Agilent J&W) in a 3:1 split mode.

#### Sensory profile

Sensory properties of cocoa liquor were evaluated by seven trained panelists (age 22 and 45), under informed consent. The panelists make part of the sensory evaluating panel of cocoa liquor and cocoa-based products from the Nataima Research center. The analysis followed the protocols established by the Sensory evaluation laboratory protocols of Nataima research center of AGROSAVIA. All methods were carried out in accordance with relevant guidelines and regulations. A Quantitative Descriptive Analysis (QDA) was applied to evaluate the flavor attributes ranges in a 0–10 scale, where 0 means absent and 10, highly pronounced or present. This research did not carry out any biomedical study or any experimentation with humans subjects. The aim was to bring light to the fermentation process to ensure the quality and homogeneity of cocoa-based products. Panelist only tastes the samples (less than 1 g), to qualify the following sensory attributes cocoa flavor, astringency, bitterness, acidity, sourness, fruity, floral, nutty, panela, caramel, and malt, managing to identify the state of fermentation of the samples and possible off-flavors conferred by inappropriate processing, such as abrupt drying or low fermentation percentages. The cocoa liquor evaluated at 45 °C was obtained after roasting at 125 °C for 15 min in a Quantik rotary toaster, removing the husk and, grinding the beans in a Kitchen brand blade processor for 20 min until a paste with a particle size of 70 microns was obtained. Duplicate samples of the liquor were evaluated in three tasting sessions to arrive at the sensory profile.

### Statistical analysis

All the analyses were carried out by triplicate and the Generalized Linear Mixed Models (GLMMs) were used to estimate fixed effects and interactions. The Backward procedure based on a 5% p-value was used to select a reasonable model for each variable. Comparisons of factor levels were made by means of Fisher’s least-squares means using Student t-statistics based on the Satterthwaite approximation (effective number of degrees of freedom). Each response variable was evaluated at the beginning of fermentation (day 0), on days 2, 3, 5 (end of fermentation) and on day 8 (drying). The liquor obtained was compared—in terms of variables associated with the aroma profile—with the unfermented cocoa in the different locations. In each model, the start of fermentation was selected as the control time to verify if the increases or decreases in time were significant according to the ‘t’ test. To verify if each model was adjusted and the normality of the data, the distribution of Pearson's residuals in each variable was evaluated, indicating that the Gaussian distribution was adequate for all variables. These models were designed using Proc GLIMMIX from the Statistical Analysis System (SAS) V. 9.4. Considering that the variables associated with the sensory analysis were not normal, the Kruskal–Wallis test was used at 5% for each profile identified in the cocoa liquor obtained at each location, using the SAS V. 9.4 Proc npar1way procedure. Finally, the Spearman correlation was applied to the sensory variables, selecting the significant relationships based on a value of P ≤ 0.05, using the SAS V. 9.4 PROC CORR procedure.

## Supplementary Information

Supplementary Information.
