# Supplementary material for: Dynamics of cocoa fermentation and its effect on quality
Source: Sci Rep. 2021 Aug 18;11:16746. doi: 10.1038/s41598-021-95703-2 (PMC8373873; doi:10.1038/s41598-021-95703-2)
Supplement: Supplementary file 1 — Supplementary Information. [file 41598_2021_95703_MOESM1_ESM.docx]

**Table S1**. LOD, LOQ, calibration curve and r^2^ for each of the compounds evaluated

| **Analytical method** | **LOD** | **LOQ** | **Calibration curve** | **(r²)** |
| --- | --- | --- | --- | --- |
| Epicatechin | 0,20 mg/g | 0,65 mg/g | Y= 0,0694 X-0,0831 | 0,992 |
| Catechin | 0,01 mg/g | 0,15 mg/g | Y=0,0684 X-0,0224 | 0,999 |
| Procyanidins | 0,50 mg/g | 1,4 mg/g | Y=14,621X+17,55 | 0,990 |
| Citric acid | 0,16% | 0,38% | Y=33,52X-0,696 | 0,990 |
| Lactic acid | 0,12% | 0,31% | Y=21,805X-0,7581 | 0,990 |
| Acetic acid | 0,16% | 0,38% | Y=22,798X-1,0436 | 0,990 |
| Mirístic acid | 0,02% | 0,04% | N/A | N/A |
| Palmític acid | 0,02% | 0,10% | N/A | N/A |
| Palmitoleic acid | 0,02% | 0,10% | N/A | N/A |
| Heptadecanoico acid | 0,02% | 0,10% | N/A | N/A |
| Stearic acid | 0,02% | 0,10% | N/A | N/A |
| Oleic acid | 0,02% | 0,10% | N/A | N/A |
| Linoleic acid | 0,02% | 0,10% | N/A | N/A |
| Araquídic acid | 0,02% | 0,04% | N/A | N/A |
| Linolenic acid | 0,02% | 0,10% | N/A | N/A |
| Araquidonic | 0,02% | 0,04% | N/A | N/A |
| Miristoleic acid | 0,02% | 0,10% | N/A | N/A |
| Pentadecanoic | 0,02% | 0,10% | N/A | N/A |
| cis-10-pentadecanoic | 0,02% | 0,10% | N/A | N/A |
| cis-10-heptadecenoic | 0,02% | 0,10% | N/A | N/A |
| theobromine | 0,45 mg/g | 1,55 mg/g | Y= 0,4439X +0,5613 | 0,999 |
| Caffeine | 0,18 mg/g | 0,55 mg/g | Y= 0,4993X+0,0687 | 0,999 |
| Reducing sugars | 0,15 % | 0,20% | Y= 1,5972X-0,0448 | 0,999 |
| Non-Reducing sugars | 0,10% | 0,17% | Y=1,3441X+0,2282 | 0,999 |
| 2.3-butanediol | 0,024 mg/L | 0,037 mg/L | N/A | N/A |
| 2,3-Dimethylpyrazine | 0,024 mg/L | 0,037 mg/L | N/A | N/A |
| Benzaldehyde | 0,024 mg/L | 0,037 mg/L | N/A | N/A |
| 2,3,5-Trimethylpyrazine | 0,024 mg/L | 0,037 mg/L | N/A | N/A |
| Phenethyl alcohol | 0,024 mg/L | 0,037 mg/L | N/A | N/A |
| Acetophenone | 0,024 mg/L | 0,037 mg/L | N/A | N/A |
| 2-Acetyl pyrrole | 0,024 mg/L | 0,037 mg/L | N/A | N/A |
| 2.3.4.5-tetramethyl pyrazine | 0,024 mg/L | 0,037 mg/L | N/A | N/A |
| linalool oxide | 0,024 mg/L | 0,037 mg/L | N/A | N/A |
| phenethyl alcohol | 0,024 mg/L | 0,037 mg/L | N/A | N/A |
| Linalool | 0,024 mg/L | 0,037 mg/L | N/A | N/A |
| Etilbenzoat | 0,024 mg/L | 0,037 mg/L | N/A | N/A |
| 2-fenilacetate | 0,024 mg/L | 0,037 mg/L | N/A | N/A |
| N | 0,10% | 0,32% | N/A | N/A |
| P | 0,001% | 0,003% | y=0,0567x+0,0172 | 0,996 |
| K | 0,001% | 0,003% | y=0,2130x+0,0226 | 0,999 |
| Ca | 0,005% | 0,017% | y=0,0333x+0,0166 | 0,999 |
| Mg | 0,001% | 0,003% | y=0,7441x+0,0403 | 0,998 |
| Na | 0,001% | 0,003% | y=0,3501x+0,0185 | 0,998 |
| S | 0,002% | 0,006% | y=0,0319x+0,0276 | 0,998 |
| Fe | 4,50 mg/kg | 15 mg/kg | y=0,0569x+0,0076 | 0,999 |
| Cu | 0,90 mg/kg | 3 mg/kg | y=0,0733x+0,0056 | 0,998 |
| Mn | 0,90 mg/kg | 3 mg/kg | y=0,1203x+0,0051 | 0,998 |
| Zin | 0,90 mg/kg | 3 mg/kg | y=0,3121x+0,0381 | 0,996 |
| B | 0,60 mg/kg | 2 mg/kg | y=0,1468x+0,0069 | 0,999 |
| Total polyphenols | 0,47924 mg acido gálico/100 g MS | 1,5974986549542 mg acido gálico/100 g MS | y=0.0032x+0.0203 | 0,998 |
